# Five years of *Trials*

**DOI:** 10.1186/1745-6215-12-248

**Published:** 2011-11-23

**Authors:** Douglas G Altman, Iain Hrynaszkiewicz, Curt D Furberg, Jeremy M Grimshaw, Peter M Rothwell

**Affiliations:** 1 Centre for Statistics in Medicine, Wolfson College Annexe, Linton Road, Oxford OX2 6UD, UK; 2 BioMed Central Ltd, 236 Gray's Inn Road, London WC1X 8HB, UK; 3 Division of Public Health Sciences, Wake Forest University School of Medicine, Medical Center Boulevard, Winston-Salem, NC 27157-1063, USA; 4 Clinical Epidemiology Programme, Ottawa Hospital Research Institute, and Department of Medicine, University of Ottawa, 501 Smyth Road, Ottawa ON K1H 8L6, Canada; 5 Nuffield Department of Clinical Neurosciences Level 6, West Wing John Radcliffe Hospital Headington Oxford OX3 9DU, UK

## Abstract

This editorial marks the launch of a special collection of articles highlighting 'Five years of *Trials' *(http://www.trialsjournal.com/series/5years). The journal's achievements on its objectives since 2006 are described and some of the challenges still ahead are outlined - in particular further innovating in the reporting of trials and the publication of negative results. The other articles in this series are examples of where *Trials *has demonstrated progress on its objectives. These include the publication of raw data, extended versions of previously published trial-related articles, descriptions of 'lessons learned', negative results, and educational articles regarding ethics and reporting bias.

## Introduction

We launched *Trials *to create a space for all types of article related to randomised trials taking advantage of the unlimited space provided by a web-based publication [1]. *Trials *is a forum for discussing both specific randomised trials as well as general issues applying to trials. Over its five years of publication, *Trials *has become recognised for its ambition to publish all trial results regardless of their outcome [2], study protocols [3], and critiques of trials published elsewhere [4]. *Trials *has also encouraged innovation in trial reporting [5,6] and stimulated debate in the medical research community [7]. In this editorial, which marks the launch of a special retrospective collection of articles highlighting 'Five years of *Trials' *(http://www.trialsjournal.com/series/5years), we discuss the journal's achievements to date, and outline some of the challenges still ahead.

The other eight articles featured in this series are examples - although there are others - of where *Trials *has demonstrated progress on its objectives since the launch of the journal. These articles cover the publication of raw data; publication of extended versions of articles previously published summarily elsewhere; reflective, lessons learned, articles; negative results of trials; and content of educational value on important issues in trial conduct and reporting, such as ethics and reporting bias, which might not have found suitable publication venues before the existence of *Trials*.

## Achievements

### Protocol publication

*"Trials will ... encourage the publication of protocols" *[1]

Recent years have seen a move to greater transparency in reporting research and especially randomised trials, most obviously in the widespread requirement for trial registration [8]. Also, five years ago evidence had begun to accumulate of wide-scale discrepancies between trial publications and what had been stated in the original trial protocol [9]. Publication of trial protocols was rarely possible in paper-based journals [10].

Increasing protocol publication is undoubtedly one of *Trials*' greatest successes. When the journal re-launched as *Trials *in 2006 we published 21 study protocols in the first year; in the first three quarters of 2011 we have already published more than 100. This growth is a significant achievement but has not been without its challenges. Publication of protocols has brought the challenge of developing guidance for their peer review. The peer-review process, and indeed whether this is routinely required for protocols, has been an ongoing discussion, and the process has been refined and updated since we launched the journal. We have aimed to promote transparency in trial reporting, by being inclusive of submitted protocols - many of which have undergone peer review during grant and ethical approval applications - but have also aimed to publish responsibly. *Trials *has rejected less than 20% of study protocols submitted to the journal, the majority of these because they fall outside of the journal's scope - for example if they describe non-randomised studies. However, approximately 5% of study protocols have been rejected because the peer reviewers deemed them to be fundamentally - methodologically - flawed. Given the substantial experience of the journal in protocol peer review and publication, and the availability of peer reviews and editors' notes openly, as pre-publication history, we intend to conduct a more detailed analysis of our experience with protocol peer-review.

We eagerly anticipate the finalisation of the SPIRIT (Standardised Protocol Items: Recommendations for Interventional Trials) checklist [11] as an opportunity to enhance our policies further. Meanwhile, four specific points for peer reviewers of study protocols to consider were added to our peer reviewer guidelines in August 2011 (see Appendix 1 and [12]). These take into account the limited changes that can pragmatically be made to a submitted study protocol without needing further ethical approval while a trial is still ongoing.

### Publication of raw data

*"We will [also] encourage authors to make available all (or some of) the raw data from the trials." *[1]

*Trials *has always encouraged authors to use the opportunities afforded by electronic publishing to include all or some of the raw data supporting the results reported in their articles (additional data files are considered integral to the associated articles, as opposed to "supplementary material" in most online publications). Technological advances have made data sharing and publication readily possible and we -perhaps naively - initially expected a reasonable proportion of authors to publish their raw data freely, the benefits of which have been widely discussed in *Trials *and other journals [13]. But while sharing trial data is widely recognised as a good idea, there are substantial cultural, legal and ethical challenges not yet comprehensively overcome in sharing clinical research data.

Despite a willingness to share data, the way is not always clear. Seeking to address some of the practical challenges of data sharing and show leadership in trial reporting, *Trials *has initiated debate [13] with the scientific community and subsequently published guidance and recommendations to increase sharing of clinical research data. These articles have included a code of conduct for data sharing and re-use [14] and detailed guidelines for authors, editors and peer reviewers on how data can be shared, and published, while maintaining patient confidentiality [15]. These guidelines have subsequently been adopted - and published - in the BMJ [16]. And in 2011 *Trials *published its first article with the primary purpose of making available for alternative analyses the individual patient data from one of the largest trials in acute stroke ever conducted, the International Stroke Trial (IST-1) [17]. This article follows the guidance developed by *Trials *and serves as an exemplar for other trialists interested in publishing their data.

Awareness of the need for transparency in all aspects of the research process seems to be increasing. Last year the US Department of Health and Human Services announced its Open Government strategy [18] and in January 2011 a consortium of 17 major funders of public health research committed to working towards increased data availability [19]. And this summer's UK government peer review enquiry called for access to raw data [20]. These are just a few examples and *Trials*, in partnership with our publisher, will remain committed to enhancing data sharing and publication.

### A reflection of previous trials - lessons learned

*"Trials will ... present commentaries on, and critiques of, trial reports published in other journals." *[1]

Much can be learned from non-completed trials. TEAM (Trial on Endovascular Aneurysm Management) was initiated in September 2006 but stopped in June 2009 after failing to reach its expected sample size [21]. Just 80 of a planned 2000 patients had been recruited. Raymond *et al*.'s account of the multi-factorial reasons - methodological, financial, cultural, ethical, bureaucratic - for the failure of the TEAM trial demonstrates the value afforded by openness regarding failures as well as successes, as designers of subsequent similar trials will be better informed. Indeed, this is one of numerous examples of *Trials *enabling the publication of articles that otherwise would probably not have existed, such as our ongoing collection on ethics issues in cluster trials [22].

### Expanded versions of previously published articles

*"Trials will consider for publication detailed, extended versions of reports of RCTs that have already been published in a conventional, short form" *[1]

Recognising the deficiencies of abbreviated article formats, we have encouraged trialists to submit substantially extended versions of articles previously published elsewhere. While expanded reports of trial results as yet remain elusive, we have published several protocols which have previously been published in summary form - in *The Lancet *[23] and *Cerebrovascular Disorders *[24]. Publication of extended reports has been supported by progressive editorial guideline development at BioMed Central regarding duplicate and overlapping publications [25].

### Challenges still ahead

Within the last five years published evidence about the impact of the CONSORT statement has supported an, albeit slow and small, improvement in the reporting of trials [26]. But many deficiencies remain - along with further challenges to overcome for *Trials*. But evidence accumulates of the widespread selective reporting of trial results, and common discrepancies between registration details and both protocols and publications have been identified [8,27,28].

### Negative results

*Trials *encourages and facilitates the publication of all scientifically sound trials - regardless of outcome. In 2009 Salarifar *et al*. reported that oral n-acetylcysteine showed no benefit, compared to an aggressive hydration protocol, in patients with diabetes mellitus and chronic kidney disease [29]. This is, however, one of a relatively small number of examples of so-called negative trials published in the last five years. The published literature over-emphasizes the positive; the reasons and motivations for reporting bias are many (for a review see [30]). The pharmaceutical industry is often singled out for suppression of results unfavourable to marketed products, although there are a number of contributing factors including, albeit inadvertently, journal editors and peer reviewers [31]. We reiterate our previous calls to make available the results of all trials, to reduce bias in the medical evidence. *Trials *will consider manuscripts previously rejected by other journals, on grounds of 'interest level' or space restrictions, and encourages authors to provide any previous peer reviews to expedite consideration by *Trials*. This approach saves authors' and peer reviewers' limited but essential time and, by making peer review more efficient, this may help reduce its hidden costs - conservatively estimated at £1.9bn globally [32].

### Innovation in trial reporting

#### Threaded publications

While *Trials *now regularly publishes the results of trials, primary trial reports are predominantly published elsewhere, and for major trials at least this is unlikely to change. To better achieve our stated aims of facilitating "*the publication of a series of linked publications from a single trial, beginning with the study protocol*"[1] BioMed Central has launched a 'threaded publications' initiative, with *Trials *as key partner journal [33]. This concept of threaded publications - clearly linking together on the web all publications regarding a specific clinical study regardless of where they are published - was floated in *The Lancet *by Chalmers and Altman in 1999 [10]. In 2011 this has begun to be put into practice, with financial incentives - discounts on publication charges - for authors to publish all protocols and results; innovative article types and journals, such as *Trials*, to accommodate all trial-related publications; and technological enhancements to the literature. Articles published in *Trials *which include a trial identifier in the abstract are now hyperlinked to the corresponding trial registration record [34]. But the concept of threaded publications is fundamentally important and so must go beyond a single journal, or indeed a single publisher, and the required innovations to make complete trial information more readily available must go further. The Editors of *Trials *are working with BioMed Central and CrossRef, the organisation founded by publishers which assigns digital object identifiers to scholarly articles to help ensure their permanence online, to develop a mechanism to implement threaded publications across multiple journals and publishers [35].

#### Update articles

Important changes to study protocols can be made in the course of a trial, which currently do not have a suitable publication format. Moreover, the conclusions or results of some studies can change after the emergence of new data. Research updates have been proposed and published elsewhere [36], but we are developing a specific format for publishing updates which gives each research or protocol update a formal, separate, citable publication.

#### Towards more structured reporting

Radical changes to how primary trial results are reported and presented in journals may yet be needed. In our editorial in 2006 we expressed the hope that we could use "the opportunities of electronic publishing to improve the reporting of randomised trials" [1]. This goal remains unfulfilled, but we aim to move forward in the next few years. There is increasing recognition that conventional journal articles often fail to provide useable information for readers [37]. One way to improve things is to impose more structure. A proposal along these lines has been made for prognosis research [38] and we hope to explore related initiatives for reports of randomised trials.

### Acknowledging our contributors

Our Editorial Board has changed substantially over the last five years, in particular by the appointment of a growing number of Associate Editors. Indeed, the continued growth and development of the journal would not have been possible without the expertise and support of our Editorial Board and peer reviewers, to whom we are very grateful.

### An evolving journal

The scope and policies of *Trials *have continued to evolve. For example we only consider study protocols for ongoing trials - which we now define as trials that are still actively recruiting patients. And with the imminent launch of our sister journal, *Systematic Reviews *(http://www.systematicreviewsjournal.com), we now encourage all systematic review products -including systematic reviews of randomised trials - to be submitted to this new publication. We are looking forward to working with the Editors of *Systematic Reviews *and past and future contributors to *Trials *over the next five years to further address the challenges of enhancing the reporting of healthcare research.

## Appendix 1

Reviewers of study protocols for *Trials *are asked to consider the following points:

1. Will the study design adequately test the hypothesis?

2. Are sufficient details provided to allow replication of the work or comparison with related analyses: if not, what is missing?

3. Is the planned statistical analysis appropriate?

4. Is the writing acceptable?

## Competing interests

DGA, CF, JG and PR are Editors-in-Chief of *Trials*; IH is an employee of BioMed Central, which publishes *Trials*.

## Authors' contributions

IH and DGA wrote the first draft of the manuscript. CF, JG and PR were involved in review and critical revision of the content before publication. All authors read and approved the final manuscript.
